# SQSTM1/p62 at the Crossroads of Autophagy, Inflammation, and Lethal Infection

**DOI:** 10.3390/cells15070652

**Published:** 2026-04-07

**Authors:** Ruoxi Zhang, Rui Kang, Daolin Tang

**Affiliations:** Department of Surgery, UT Southwestern Medical Center, Dallas, TX 75390, USA

**Keywords:** SQSTM1/p62, autophagy, sepsis, inflammation, damage-associated molecular pattern (DAMP)

## Abstract

Sequestosome 1 (SQSTM1, also known as p62) has emerged as a multifunctional signaling adaptor that bridges autophagy, proteostasis, and inflammation. In this review, we discuss the molecular mechanisms by which SQSTM1 regulates selective autophagy and immune signaling pathways, and how its dynamic modulation shapes host responses during sepsis. We highlight the tissue-specific roles of SQSTM1 in sepsis-associated injury across major organs—including the liver, kidney, heart, lung, brain, and skeletal muscle—and explore its function as a damage-associated molecular pattern (DAMP) in the extracellular milieu. Recent studies implicate extracellular SQSTM1 in metabolic reprogramming and pro-inflammatory cytokine production via INSR signaling, supporting its classification as a novel DAMP and potential therapeutic target. We conclude a stage- and compartment-specific model for SQSTM1 during sepsis: its transition from a protective intracellular autophagy mediator in the early stage to a pathological extracellular DAMP in late stage. Furthermore, we discuss the translational relevance of pharmacological agents that modulate SQSTM1 levels or activity to restore immune balance and organ homeostasis. A better understanding of SQSTM1’s dual roles in immune activation and resolution could open new avenues for precision therapies in sepsis.

## 1. Introduction

Innate immune cells—including macrophages, monocytes, and neutrophils—form the first line of defense against invading pathogens. These cells sense microbial products and host-derived danger signals through pattern-recognition receptors (PRRs) that detect pathogen-associated molecular patterns (PAMPs) and damage-associated molecular patterns (DAMPs) [1]. PAMPs encompass microbial components such as bacterial DNA, RNA, and lipopolysaccharide (LPS), a major constituent of Gram-negative bacterial membranes. DAMPs, in contrast, are endogenous molecules released during cellular stress or injury, including proteins such as high-mobility group box 1 (HMGB1) and non-protein signals such as extracellular DNA and ATP [2]. Engagement of PRRs activates downstream signaling cascades that orchestrate inflammatory responses essential for pathogen clearance and tissue repair. However, sustained or excessive activation can lead to dysregulated inflammation, culminating in tissue injury, multi-organ failure, and death—a pathological continuum that defines sepsis and septic shock. Although PAMPs and DAMPs remain attractive therapeutic targets, clinical outcomes are still unsatisfactory, reflecting the complex and heterogeneous nature of lethal infection [3].

Sequestosome 1 (SQSTM1, also known as p62) was originally identified as a binding partner of the Src family tyrosine kinase LCK [4]. It is now recognized as a multifunctional signaling adaptor that integrates diverse cellular processes, most notably selective autophagy, inflammatory signaling, and oxidative stress responses. A hallmark of SQSTM1 is its ability to function as an autophagy receptor by linking polyubiquitinated cargo to microtubule-associated protein 1 light chain 3 (MAP1LC3/LC3)-positive autophagosomes, thereby directing substrates for lysosomal degradation [5]. Through this mechanism, SQSTM1 participates in multiple forms of selective autophagy—including aggrephagy, mitophagy, lysophagy, xenophagy, lipophagy, and clockophagy—each of which contributes to cellular homeostasis [6,7]. Disruption of these pathways, whether through impaired or excessive autophagy, can amplify inflammatory signaling and sensitize tissues to injury [8]. Beyond its degradative roles, SQSTM1 acts as a central node in inflammatory and stress-response pathways. It modulates nuclear factor κB (NF-κB) activation, regulates redox balance, and interfaces with several regulated cell death programs [6,7,9]. Through these diverse functions, SQSTM1 sits at a critical intersection where selective autophagy, inflammation, and proteostasis converge—processes that are profoundly altered during infection and sepsis.

In this review, we summarize the molecular mechanisms through which SQSTM1 governs autophagy, shapes inflammatory responses, and influences host susceptibility to infection. We highlight a stage- and compartment-specific model in which intracellular SQSTM1-mediated autophagic homeostasis is predominantly protective in early infection, whereas impaired flux, SQSTM1 accumulation, and extracellular SQSTM1 signaling drive inflammatory amplification and organ injury in late-stage lethal infection. We also discuss its translational potential as a therapeutic target in sepsis and its complications.

## 2. Structure and Regulation of SQSTM1

SQSTM1 is a 62 kDa protein that is primarily localized in cytoplasmic condensates and widely expressed across various cell types and tissues. Its expression is highly inducible in response to various stress conditions and is tightly regulated at both transcriptional and post-translational levels [10].

SQSTM1 transcription is activated in response to diverse cellular stresses. Amino acid deprivation or endoplasmic reticulum (ER) stress induces SQSTM1 expression via activating transcription factor 4 (ATF4) [11]. Under nutrient starvation, transcription factor EB (TFEB), a master regulator of lysosomal biogenesis and autophagy, translocates to the nucleus and activates SQSTM1 transcription [12,13]. Inflammatory conditions activate NF-κB transcription factor, which also enhances SQSTM1 expression [14]. In oxidative stress, NFE2 like BZIP transcription factor 2 (NFE2L2, also known as NRF2) binds to the antioxidant response element (ARE) in the SQSTM1 promoter and promotes its transcription. In turn, accumulated SQSTM1—especially under autophagy impairment—binds to KEAP1 (Kelch-like ECH-associated protein 1), the negative regulator of NFE2L2, leading to reduced proteasomal degradation of NFE2L2 [15]. This establishes a positive feedback loop that reinforces antioxidant defense mechanisms.

SQSTM1 activity is finely tuned through post-translational modifications, including phosphorylation [16], ubiquitination [17], and acetylation [18]. For example, phosphorylation at serine 403 (S403) by kinases TANK binding kinase 1 (TBK1) or Unc-51 like autophagy activating kinase 1 (ULK1) increases its affinity for polyubiquitin chains, thereby promoting the selective autophagic clearance of ubiquitinated proteins [16]. Conversely, phosphorylation of S403 by mitogen-activated protein kinase kinase kinase 7 (MAP3K7, also known as TAK1) and heat shock transcription factor 1 (HSF1) impairs SQSTM1’s ability to mediate cargo degradation [19,20].

**Figure 1:**
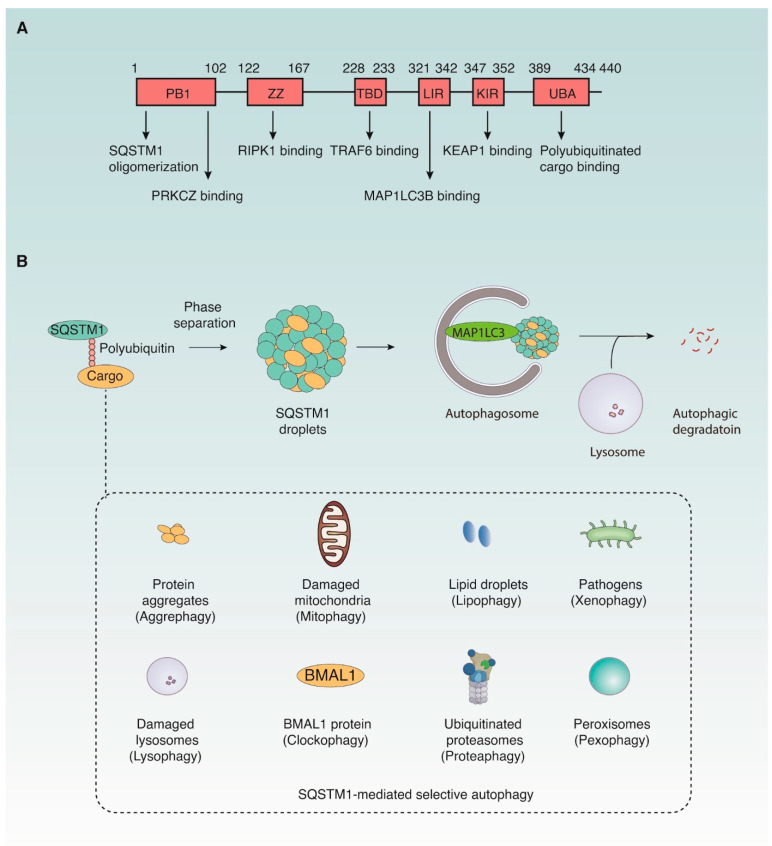
SQSTM1 structure and its mediated selective autophagy. (**A**) SQSTM1 Structure. The human SQSTM1 protein consists of 440 amino acids and contains several conserved domains that mediate interactions with both ubiquitinated substrates and signaling proteins. The PB1 domain enables oligomerization and interaction with kinases, such as PRKCZ. The ZZ domain binds RIPK1 and TRAF6 to regulate inflammatory signaling. The KIR motif interacts with KEAP1 to modulate oxidative stress. The LIR motif binds MAP1LC3B to mediate autophagosome recruitment. The UBA domain recognizes polyubiquitinated cargo. Through these domain-based interactions, SQSTM1 functions as both a signaling hub and a selective autophagy receptor. (**B**) SQSTM1-mediated selective autophagy through phase separation and cargo recognition. SQSTM1 binds polyubiquitinated cargo and undergoes liquid–liquid phase separation to form membrane-less condensates (droplets) that concentrate autophagic substrates. These SQSTM1 droplets are subsequently engulfed by autophagosomes via interaction with MAP1LC3 and delivered to lysosomes for degradation. SQSTM1 facilitates selective autophagy of diverse cargo types, including protein aggregates (aggrephagy), damaged mitochondria (mitophagy), lipid droplets (lipophagy), intracellular pathogens (xenophagy), damaged lysosomes (lysophagy), the core circadian protein BMAL1 (clockophagy), ubiquitinated proteasomes (proteaphagy), and peroxisomes (pexophagy). This process is essential for maintaining cellular proteostasis and stress adaptation during infection and inflammation.

SQSTM1 contains several conserved domains that mediate its interactions with ubiquitinated substrates and signaling proteins, enabling it as a signal hub and selective autophagy receptor [6,9] (Figure 1A). The Phox1 and Bem1p (PB1) domain (1–102) at the N-terminus facilitates oligomerization of SQSTM1, which is critical for its function and localization [21]. It also interacts with atypical protein kinase C zeta (PRKCZ, also known as PKCζ), contributing to oxidative responses and NF-κB-mediated inflammatory responses [22,23]. The ZZ-type zinc finger (ZZ) domain (122–167) recognizes N-terminally arginylated proteins for autophagic degradation and recruits receptor interacting serine/threonine-protein kinase 1 (RIPK1) for the necrosome assembly [24,25]. The ubiquitin-associated (UBA) domain (389–434) at the C-terminus recognizes polyubiquitinated protein cargoes for autophagic degradation and mediates phase separation of SQSTM1 [26].

The middle intrinsically disordered region (IDR, 168–388) contains several functional motifs (Figure 1A). For example, TRAF binding domain (TBD, 228–233) links SQSTM1 to tumor necrosis factor receptor-associated factor 6 (TRAF6), promoting downstream activation of NF-κB signaling [27]. Keap1-interacting region (KIR, 347–352) regulates the KEAP1-NFE2L2 antioxidant pathway [28,29], whereas LC3 interacting region (LIR, 321–342) is responsible for binding with autophagy-related protein 8 (ATG8) family, a group of ubiquitin-like proteins that play central roles in autophagy, particularly in autophagosome formation, cargo recognition, and membrane dynamics [30]. In mammals, the ATG8 family is subdivided into two main subfamilies: MAP1LC3 (including MAP1LC3A, MAP1LC3B, and MAP1LC3C) and GABA type A receptor-associated protein (GABARAP, including GABARAP, GABARAPL1, and GABARAPL2) subfamily. In addition, SQSTM1 interacts with regulatory associated protein of MTOR complex 1 (RPTOR), a core component of the mechanistic target of rapamycin kinase (MTOR)-orchestrated nutrient-sensing complex, through its ZZ and TBD domains [31]. This interaction links SQSTM1 to MTOR signaling and metabolic regulation.

Despite extensive characterization, several unanswered questions remain regarding the structural and regulatory complexity of SQSTM1. For example, how distinct post-translational modifications of SQSTM1 are temporally and spatially coordinated under different stress conditions remains poorly understood. Moreover, the precise mechanisms by which SQSTM1 phase separation is dynamically regulated to control selective autophagy versus signal transduction require further investigation.

## 3. SQSTM1 and Autophagy

Cellular proteostasis is maintained by two major degradation pathways: the ubiquitin-proteasome system (UPS) and autophagy. Autophagy is responsible for the removal of long-lived proteins, insoluble protein aggregates, dysfunctional organelles, and invaded pathogens [32]. In contrast, the UPS mainly targets short-lived and misfolded soluble proteins for proteasomal degradation [33]. SQSTM1 functions at the interface of these two systems by acting as a selective receptor that facilitates the delivery of polyubiquitinated proteins to either the autophagosome or the proteasome [30,34], thus serving as a critical hub in cellular protein quality control.

Autophagy is a tightly regulated catabolic process in which autophagy receptors, such as SQSTM1, recognize specific cargoes and mediate their sequestration into autophagosomes for lysosomal degradation [35]. SQSTM1 was the first autophagy receptor identified in mammalian cells and remains one of the most extensively studied [30]. It contains a C-terminal UBA domain that binds polyubiquitinated cargoes and a LIR that anchors these substrates to the autophagosomal membrane via interaction with ATG8 family. SQSTM1 undergoes liquid–liquid phase separation to form dynamic, membrane-less condensates—referred to as SQSTM1 bodies or droplets—that concentrate autophagic cargoes and serve as platforms for autophagosome biogenesis and signaling [36,37] (Figure 1B).

SQSTM1 mediates multiple forms of selective autophagy depending on the nature of its cargo, including aggrephagy (protein aggregates) [38], mitophagy (damaged mitochondria) [39], lipophagy (lipid droplets) [40], xenophagy (pathogens) [41], lysophagy (damaged lysosomes) [42], clockophagy (basic helix-loop-helix ARNT like 1 [BMAL1]) [43], proteaphagy (ubiquitinated proteasomes) [44], and pexophagy (peroxisomes) [45] (Figure 1B). In addition to functioning as an autophagy receptor, SQSTM1 itself is degraded by autophagy, which provides a mechanism to prevent excessive autophagic activation [46]. Thus, SQSTM1 is widely used as a marker of autophagic flux—its reduction typically indicates autophagic activation, while accumulation suggests impaired autophagy [47]. However, interpretation can be confounded by stress-induced transcriptional upregulation of SQSTM1, as described earlier.

The UPS is a highly dynamic and reversible degradation pathway that governs protein homeostasis via a cascade of enzymatic reactions [48]. Proteins are tagged for degradation by covalent attachment of ubiquitin through the sequential action of E1 (activating), E2 (conjugating), and E3 (ligating) enzymes [49]. This ubiquitination directs proteins to the 26S proteasome, where they are recognized and degraded. Deubiquitinating enzymes (DUBs) can reverse this modification, thereby modulating proteasomal degradation. SQSTM1 not only binds polyubiquitinated proteins via its UBA domain but also directly interacts with the 19S regulatory particle of the proteasome through its PB1 domain, particularly with proteasome 26S subunit ubiquitin receptor, non-ATPase 2 (PSMD2) and proteasome 26S subunit ubiquitin receptor, non-ATPase 4 (PSMD4) subunits , to facilitate proteasomal delivery [50]. Furthermore, SQSTM1-rich phase-separated condensates can recruit active proteasomes to selectively degrade nuclear proteins and unassembled subunits [51]. Interestingly, SQSTM1 itself is regulated by the UPS. For example, the E3 ubiquitin ligase parkin (PRKN), a key regulator of mitophagy, binds to the PB1 domain of SQSTM1 and ubiquitinates it at lysine 13 (K13), targeting it for proteasomal degradation [17].

SQSTM1 links autophagy and the UPS to ensure proteostasis under stress. For instance, persistent proteasome inhibition or ubiquitin overload leads to SQSTM1 accumulation and activation of autophagy as a compensatory mechanism, whereas autophagy inhibition leads to SQSTM1 accumulation, which can sequester ubiquitinated proteins into non-degradable aggregates and impair their proteasomal degradation [52]. SQSTM1-mediated proteaphagy is particularly important for clearing damaged or inactive proteasomes during nutrient starvation or stress, thereby maintaining proteasome homeostasis [44].

In summary, precise regulation of SQSTM1 is essential for sustaining proteostasis, particularly under pathological or stress conditions. However, key questions remain regarding how SQSTM1 coordinates its dual roles in autophagy and the UPS, how post-translational modifications influence its cargo specificity, and how SQSTM1-containing condensates are dynamically regulated in different subcellular compartments.

## 4. SQSTM1 as a Signaling Hub During Inflammation

**Figure 2:**
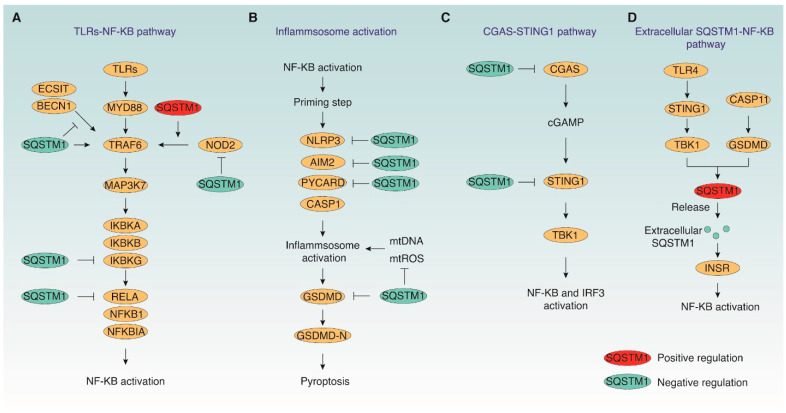
Dual regulatory roles of SQSTM1 in inflammatory signaling and innate immune activation. (**A**) SQSTM1 modulates Toll-like receptors (TLRs) and NOD2 signaling pathways by functioning as a signaling scaffold while also promoting the degradation of selected pathway components to limit excessive activation. It facilitates NF-κB activation through interaction with TRAF6 and MAP3K7 while also targeting components, such as TRAF6, IKBKG in IKK complex (inhibitor of nuclear factor kappa B kinase subunit alpha [IKBKA], IKBKB, and IKBKG), and RELA in NF-κB complex (RELA, nuclear factor kappa B subunit 1 [NFKB1], and NFKB inhibitor alpha [NFKBIA]), for autophagic degradation to restrain inflammation. SQSTM1 also negatively regulates ECSIT–BECN1–TRAF6 signaling complexes. (**B**) SQSTM1 restrains inflammasome signaling by facilitating mitophagy and the selective autophagic clearance of inflammasome-related components. SQSTM1 suppresses inflammasome activation and active pore-forming N-terminal fragment of GSDMD (GSDMD-N) by targeting inflammasome components (NLRP3, AIM2, PYCARD, CASP1) and GSDMD for autophagic degradation, and by promoting mitophagy to reduce mitochondrial DAMPs (mtDNA, mtROS) that drive inflammasome priming and activation. (**C**) SQSTM1 attenuates cGAS–STING1 signaling through autophagic turnover of pathway components. SQSTM1 attenuates cGAS–STING1–TBK1–IRF3 signaling by mediating degradation of both cGAS and STING1, thereby limiting type I interferon and NF-κB responses. (**D**) Extracellular SQSTM1 acts as a DAMP-like mediator to amplify immunometabolic activation and cytokine production. SQSTM1 is released extracellularly upon TLR4–STING1–TBK1 activation or CASP11–GSDMD-mediated membrane rupture. Extracellular SQSTM1 acts as a DAMP by binding the insulin receptor (INSR) on immune cells, promoting glycolytic reprogramming and NF-κB–driven cytokine production.

SQSTM1 plays a dual and context-dependent role in regulating inflammation by functioning as both a signaling adaptor and a mediator of proteostasis (Figure 2). As a signaling scaffold, SQSTM1 modulates key inflammatory pathways, particularly the NF-κB signaling axis, which governs the expression of numerous pro-inflammatory cytokines and immune regulators [7]. SQSTM1 facilitates the assembly of signaling complexes by directly interacting with TRAF6, a pivotal E3 ubiquitin ligase involved in Toll-like receptor (TLR)-mediated NF-κB activation. This interaction links TRAF6 with the TLR adaptor MYD88 innate immune signal transduction adaptor (MYD88) and the upstream kinase MAP3K7 [53]. This interaction facilitates K63-linked polyubiquitination of TRAF6, allowing its recruitment and activation of downstream inhibitor of nuclear factor kappa B kinase subunit beta (IKBKB), which in turn promotes NF-κB translocation into the nucleus to induce transcription of pro-inflammatory cytokines such as TNF and IL6 [23,54]. Furthermore, SQSTM1–TRAF6 interactions enhance the association with cytosolic pattern recognition receptor NOD2 (nucleotide binding oligomerization domain containing 2), amplifying NOD2–NF-κB signaling [55].

Paradoxically, overexpression of SQSTM1 can suppress inflammation by disrupting TRAF6 signaling. It inhibits the TRAF6-ECSIT signaling integrator (ECSIT) and TRAF6-Beclin 1 (BECN1) interactions, which are essential for TLR4-mediated NF-κB activation, thereby preventing excessive inflammatory responses [56,57]. In addition, oxidative stress-induced reactive oxygen species (ROS) promote the formation of SQSTM1-ubiquitin-positive aggresome-like induced structures (ALIS), which can act as pro-inflammatory stimuli if not efficiently cleared [58], positioning SQSTM1 as a redox-sensitive signaling molecule.

Beyond its role in signal propagation, SQSTM1 contributes to inflammation resolution through proteostatic mechanisms by mediating the autophagic degradation of inflammatory signaling components (Figure 2). SQSTM1 suppresses NF-κB signaling by targeting proteins such as RELA/p65 (proto-oncogene, NF-KB subunit) and IKBKG/IKKγ (inhibitor of nuclear factor kappa B kinase regulatory subunit gamma) for degradation [59,60]. It also restrains inflammasome activation by promoting mitophagy, thereby removing mitochondrial DAMPs, including mtDNA and mitochondrial ROS, which are known triggers of inflammasome assembly [61]. SQSTM1 recognizes damaged mitochondria for autophagic degradation in macrophages undergoing PRKN-mediated ubiquitination [14]. Moreover, SQSTM1 directly targets key inflammasome components—such as NLR family pyrin domain containing 3 (NLRP3), PYD and CARD domain containing (PYCARD, also known as ASC), absent in melanoma 2 (AIM2), and gasdermin D (GSDMD)—for selective autophagic degradation [62,63,64], limiting excessive interleukin 1 beta (IL1B) production and sterile inflammation. This process is facilitated by regulatory proteins such as immunity related GTPase M (IRGM) [65], galectin 9 (LGALS9) [66], and TRIM family E3 ligases (e.g., tripartite motif containing 11 [TRIM11]), which enhance ubiquitination and SQSTM1 recruitment [67].

SQSTM1 also collaborates with other selective autophagy receptors, including NBR1 autophagy cargo receptor (NBR1), tax1 binding protein 1 (TAX1BP1), and calcium binding and coiled-coil domain 2 (CALCOCO2), to mediate degradation of components in the cyclic GMP-AMP synthase (CGAS)-stimulator of interferon response CGAMP interactor 1 (STING1) pathway, a key driver of type I interferon responses [68,69]. For instance, autophagic clearance of CGAS and STING1 by SQSTM1 and its cofactors restricts excessive inflammatory signaling. Interestingly, S-palmitoylation of NOD2 by the palmitoyltransferase ZDHHC5 (zinc finger DHHC-type palmitoyltransferase 5) impairs SQSTM1-mediated NOD2 degradation, sustaining NF-κB activation [70]. Conversely, cleavage of SQSTM1 by caspase 1 (CASP1) limits its activity in autophagy, serving as a negative feedback loop to balance immune responses [71]. Together, these findings illustrate that SQSTM1 fine-tunes inflammation through an intricate balance between signaling activation and proteostatic restraint.

Recent studies reveal that SQSTM1 can also act extracellularly as a pro-inflammatory mediator (Figure 2). In LPS-treated macrophages and monocytes, SQSTM1 can be actively secreted via TLR4-STING1-TBK1 pathway-mediated non-classical secretion lysosomes. Mechanistically, TBK1 phosphorylates the Ser403 site on SQSTM1, which causes SQSTM1 accumulation in autolysosomes and subsequent secretion through mucolipin TRP cation channel 1 (MCOLN1, also known as TRPML1)-mediated secretory lysosomes. Simultaneously, cytosolic LPS activates caspase 11 (CASP11), resulting in the cleavage of GSDMD and subsequent plasma membrane rupture, leading to passive release of SQSTM1 [72]. Once in the extracellular space, SQSTM1 binds to the insulin receptor (INSR) on immune cells, triggering aerobic glycolysis and NF-κB–dependent cytokine production [72]. Given that intracellular SQSTM1 promotes the selective autophagic degradation of inflammasome components and GSDMD for preventing uncontrolled pyroptosis (as we discussed above), SQSTM1 links pyroptosis to both intracellular regulation of inflammasome activity and extracellular propagation of inflammation.

SQSTM1 also functions as an atypical member of atypical protein kinase C (PKC) family and interacts with PRKCZ, a downstream mediator of the insulin-INSR pathway [73]. This interaction enhances oxidase activity and further activates the TRAF6–NF-κB axis [22,23]. Given its role in oxidative stress signaling, it is conceivable that extracellular SQSTM1–INSR engagement leads to formation of a PRKCZ–TRAF6 complex that drives NF-κB-mediated inflammation. In parallel, extracellular SQSTM1 can also signal through the advanced glycosylation end product-specific receptor (AGER, also known as RAGE) instead of INSR in pancreatic acinar cells, thereby enhancing ferroptosis, a form of oxidative stress-induced cell death, and acute pancreatitis [74]. These findings support the view that extracellular SQSTM1 acts as a DAMP with receptor- and cell type-dependent inflammatory consequences.

Collectively, these insights establish SQSTM1 as a nodal signaling hub that integrates inflammatory pathways, autophagy, proteostasis, and redox signaling. Its dual role—amplifying or restraining inflammation depending on context—underscores the importance of precise regulatory control over SQSTM1 activity in immunity and inflammatory disease.

## 5. Tissue-Specific Roles of SQSTM1 in Infection

Studies in patients with sepsis have revealed significant alterations in SQSTM1 expression and systemic regulatory disruption [72,75,76], suggesting that SQSTM1 may serve as a hallmark feature of lethal infection. In this section, we summarize the emerging roles of SQSTM1 across multiple organs commonly affected during pathogen infection—including the liver, kidney, heart, skeletal muscle, brain, and lung. We also highlight its potential utility as a diagnostic or prognostic biomarker and discuss pharmacological strategies targeting SQSTM1-mediated pathways.

### 5.1. Liver

The liver plays a dual role in pathogen infection, functioning both as a critical immunometabolic organ that clears pathogens and produces inflammatory mediators, and as a frequent target of lethal infection-related injury [77]. Hepatic dysfunction often arises early during infection and significantly contributes to the development of multiple organ failure. Emerging evidence implicates SQSTM1 in regulating autophagy, inflammatory signaling, and hepatocyte survival during infection-induced liver injury.

In experimental models of infection-induced liver injury, dynamic alterations in hepatic SQSTM1 expression have been closely associated with the severity and progression of organ damage. In cecal ligation and puncture (CLP)-induced sepsis murine models, impaired autophagic flux leads to the accumulation of SQSTM1 and MAP1LC3B in hepatocytes [78,79,80,81]. Similarly, LPS administration results in blockade of late-stage autophagy, reflected by elevated MAP1LC3B-II and SQSTM1 levels, contributing to acute hepatic injury [82]. Immunofluorescence analyses have confirmed increased SQSTM1 expression in hepatic parenchyma following LPS exposure [83]. Together, these findings suggest that defective autophagy, accompanied by SQSTM1 buildup, is a hallmark feature of infection-induced liver pathology.

Mechanistic studies further support a protective role of autophagy in infection-associated liver injury. Liver-specific deletion of *Atg5* exacerbates mitochondrial damage, increases ROS production, accelerates hepatocyte apoptosis, and shortens survival in CLP mice [84]. LPS-induced ROS generation also triggers lysosomal membrane permeabilization and lysosome-dependent cell death in rat hepatocytes, reinforcing the importance of autophagy in mitigating oxidative stress and maintaining mitochondrial integrity [85].

Therapeutic strategies targeting SQSTM1-regulated autophagy pathways have shown promise in preclinical models. The selective α2-adrenoceptor agonist, dexmedetomidine, protects against CLP-induced liver injury by enhancing autophagy through activation of the protein kinase AMP-activated catalytic subunit alpha 1 (PRKAA1, also known as AMPK)-sirtuin 1 (SIRT1) signaling pathway, which further reduces hepatic inflammation [79]. This protection correlates with the timely degradation of SQSTM1 and normalization of autophagy markers, while the SIRT1 inhibitor reversed the effect of dexmedetomidine on autophagy flux and SQSTM1 expression in vitro [79]. Similarly, GTS-21, a selective α7 nicotinic acetylcholine receptor (α7 nAChR) agonist, attenuates liver damage by promoting autophagy and SQSTM1 clearance, while dampening proinflammatory cytokine production [81]. Natural antioxidants such as genipin, malvidin, and anemoside B4 also restore hepatic autophagic flux and SQSTM1 degradation, inhibit NLRP3 inflammasome activation, and improve survival in CLP models [78,80,86] (Table 1). In contrast, chloroquine—a known autophagy inhibitor—abolishes these protective effects, underscoring the importance of SQSTM1 clearance as a functional indicator of autophagy and hepatic resilience [78].

Collectively, these findings establish SQSTM1 as a key regulator of hepatocellular homeostasis during infection, linking autophagy, inflammation, and cell death. Targeting SQSTM1-mediated pathways holds translational potential for treating infection-induced liver dysfunction. Nevertheless, most of the available evidence is derived from CLP or LPS rodent models. The interspecies differences should be considered when extrapolating preclinical findings to clinical translation.

### 5.2. Kidney

Infection-induced acute kidney injury (iAKI), commonly presenting in the context of sepsis-associated AKI (SAKI), is a common and serious complication of systemic infection, contributing substantially to morbidity and mortality in critically ill patients [106]. Autophagy plays a protective role in the kidney during infection by removing damaged mitochondria and suppressing inflammasome activation. However, infection often disrupts this balance, resulting in impaired autophagic flux and exacerbated renal dysfunction [107].

In CLP-induced iAKI mouse models, BECN1 and MAP1LC3B levels are increased following CLP administration, peaking at 8 h and returning to baseline by 24 h. In contrast, SQSTM1 levels show an inverse pattern, reaching a nadir at 8 h before increasing thereafter [108]. Similar trends have been observed in LPS-induced iAKI mouse models, where both protein and mRNA levels of SQSTM1 decrease initially (4–8 h) and subsequently rise at later time points (12–24 h) [109]. These dynamic changes suggest that autophagy is transiently activated in the early phase of iAKI but subsequently declines, leading to SQSTM1 accumulation as autophagic flux becomes impaired.

These patterns highlight SQSTM1 as a functional marker of autophagic flux and disease progression in iAKI. Clinical data support this view: increased SQSTM1 accumulation in renal tissues and peripheral blood mononuclear cells (PBMCs) from sepsis patients correlates with the severity of kidney injury and may serve as a potential biomarker for monitoring disease progression and therapeutic response [75,110].

SQSTM1 modulates the delicate balance between autophagy, oxidative stress, and inflammation in infected kidneys. For instance, bone mesenchymal stem cell–derived exosomes (BMSCs-Exo) enhance autophagic activity in iAKI by increasing MAP1LC3B and phosphorylated PRKAA1 while decreasing SQSTM1 levels. These exosomes improve cell viability and exert anti-inflammatory and anti-apoptotic effects in LPS-treated HK-2 cells. Comparable renoprotective effects are observed with the autophagy inducer rapamycin, whereas the autophagy inhibitor 3-methyladenine (3-MA) partially abolishes these benefits [89].

Infection-induced AKI is also characterized by elevated expression of triggering receptor expressed on myeloid cells 1 (TREM1), an amplifier of inflammatory responses triggered by bacterial or fungal infection. Overexpression of TREM-1 promotes apoptosis and inhibits autophagy in HK-2 cells, accompanied by increased SQSTM1 accumulation [111] (Table 1). These findings support the role of SQSTM1-mediated autophagic degradation in counteracting oxidative and inflammatory injury.

Targeting cell death pathways also affects SQSTM1 turnover. Treatment with necrostatin-1, an inhibitor of RIPK1-mediated necroptosis, enhances autophagosome–lysosome fusion, promotes SQSTM1 degradation, reduces tubular epithelial apoptosis, and improves renal function in CLP-induced infection models [87]. Conversely, in LPS-induced AKI, activation of receptor-interacting protein kinase 3 (RIPK3) blocks autophagy by inhibiting TFEB, a master regulator of lysosomal biogenesis, leading to SQSTM1 accumulation. RIPK3 inhibition restores TFEB nuclear translocation, normalizes autophagic flux, and facilitates SQSTM1 degradation [112].

Pharmacological agents that modulate SQSTM1-dependent autophagy show therapeutic potential in infection-associated kidney injury. For example, recombinant erythropoietin (rhEPO) activates the PRKAA1–SIRT1 axis, enhancing autophagy and promoting SQSTM1 clearance, thereby alleviating renal injury in LPS-treated mice [108,113]. Likewise, zinc supplementation improves SAKI outcomes by stabilizing sirtuin 7 (SIRT7)-PRKN interactions, leading to decreased SQSTM1 accumulation and restored mitophagy [88]. Small-molecule activators of the SQSTM1-ZZ domain also promote mitophagy and immunometabolic reprogramming, resulting in reduced renal inflammation [114]. In contrast, inhibition of autophagy using 3-MA exacerbates kidney damage in infection models [115] (Table 1).

In summary, the SQSTM1 accumulation indicates impaired autophagic clearance and correlates with injury severity, while its timely clearance confers renal protection. Therefore, therapeutic strategies that enhance SQSTM1 turnover through restoration of autophagy may provide an approach for the prevention or treatment of iAKI. However, evidence mainly comes from rodent models, with limited confirmation in patient samples. Differences between murine and human renal responses, together with the heterogeneity of sepsis in clinical settings, highlight the need for more robust patient-based validation.

### 5.3. Heart

Infection-induced myocardial dysfunction also contributes to infection-related mortality and is characterized by metabolic reprogramming, oxidative stress, and mitochondrial dysfunction [116]. Experimental models of sepsis consistently demonstrate activation of autophagy and concurrent degradation of SQSTM1 in cardiac tissue. Both LPS and CLP models show increased expression of autophagy markers such as BECN1 and MAP1LC3B, accompanied by decreased SQSTM1 levels, indicative of active autophagic flux [117,118,119].

During infection, autophagy plays a protective role in cardiomyocytes by eliminating damaged mitochondria and misfolded proteins, with SQSTM1 functioning as a key autophagy receptor in this process [120]. However, when autophagic blockade leads to SQSTM1 accumulation, which exacerbates mitochondrial damage, apoptosis, and progressive cardiac dysfunction.

In late-stage LPS-induced cardiomyopathy, expression of PPARG coactivator 1 alpha (PPARGC1A, also known as PGC1A)—a regulator of mitochondrial biogenesis—is suppressed [121]. Overexpression of PPARGC1A restores SQSTM1-dependent autophagy, preserves mitochondrial integrity, and reduces LPS-induced cardiomyocyte apoptosis. Similarly, uncoupling protein 2 (UCP2), a regulator of oxidative stress and mitochondrial membrane potential, contributes to infection-induced cardiac dysfunction by inhibiting autophagy [122]. UCP2 inhibition promotes SQSTM1-mediated autophagy and improves cardiac function and survival in CLP-treated mice. Additionally, knockout of miR-22 alleviates CLP-induced myocardial injury by enhancing autophagy, reducing SQSTM1 levels, and inhibiting apoptosis [123].

Restoration of SQSTM1-dependent autophagy has shown cardioprotective effects in various models of infection-induced myocardial injury. For instance, the GLP-1 analog semaglutide activates PRKAA1 signaling, promoting SQSTM1 degradation, reducing inflammation, and improving cardiac function in CLP mice [95]. Similarly, dexmedetomidine treatment activates the PRKAA1-SIRT1 pathway to stimulate autophagic flux, reduce SQSTM1 accumulation, and improve myocardial histopathology and survival [79]. Rapamycin further enhances autophagy in septic hearts, offering protective effects against myocardial dysfunction [119]. Several antioxidants, including carvacrol, high-dose vitamin C, and thymoquinone, exert cardioprotective effects by restoring autophagy and promoting SQSTM1 clearance [93,94,96].

Despite these benefits, excessive autophagy may contribute to myocardial injury under certain conditions. For instance, prolonged LPS exposure (24 h) results in increased cardiomyocyte death, lactate dehydrogenase release, and malondialdehyde production. These effects are associated with excessive activation of protein kinase C beta (PRKCB) and enhanced autophagy, as indicated by increased MAP1LC3B and decreased SQSTM1 expression [91]. The anti-inflammatory drug, remifentanil, protects cardiomyocytes against LPS-induced oxidative injury by downregulating PRKCB activity, thereby suppressing autophagy and preserving SQSTM1 levels [91]. Similarly, the PRKCB inhibitor CGP 53353 attenuates LPS-induced cytotoxicity by reducing SQSTM1-mediated autophagy [124]. Inhibiting autophagy with 3-MA produces comparable cardioprotective effects, further suggesting a detrimental role of uncontrolled autophagy in energy-deprived cardiomyocytes [91,124].

Regulation of autophagy via non-coding RNAs is also implicated in myocardial protection. Overexpression of miR-214-3p activates the AKT serine/threonine kinase (AKT)–MTOR pathway, suppressing autophagy and SQSTM1 degradation, and thus alleviating myocardial dysfunction in CLP-induced infection [125]. Similarly, sodium hydrosulfide (NaHS) protects cardiomyocytes from LPS-induced injury by upregulating miR-133a-3p, which inhibits SQSTM1 degradation and autophagy, restoring intracellular ATP levels [92] (Table 1).

Collectively, the expression of SQSTM1 reflects the status of autophagic flux and myocardial injury. SQSTM1-dependent autophagy shows cardioprotective effects against lethal infection, yet its excessive induction, driven by PRKCB activation, results in myocardial cytotoxicity and subsequent myocardial injury. Pharmacological modulation of SQSTM1 degradation pathways offers promising strategies to mitigate infection-triggered myocardial inflammation. Current insights rely largely on experimental sepsis models. Given the variability in cardiac responses across species, further studies in human tissues and large patient cohorts are required to clarify the translational significance of SQSTM1 in infectious cardiomyopathy.

### 5.4. Lung

Acute lung injury (ALI) and its severe form, acute respiratory distress syndrome (ARDS), represent early and prominent manifestations of infection-related organ failure and remain among the leading causes of mortality in septic patients [3]. Clinical observations highlight the diagnostic and prognostic relevance of SQSTM1 in septic lung complications. Elevated serum levels of SQSTM1 have been reported in patients with infection-associated ARDS, with correlations to prolonged ICU stay, increased disease severity, and higher mortality [110]. This reinforces the translational significance of SQSTM1 as a circulating biomarker for monitoring ARDS progression in during severe infection.

In preclinical models of infection-induced ALI, SQSTM1 upregulation is consistently associated with impaired autophagic flux and defective protein clearance. In CLP-induced lung injury, increased SQSTM1 accumulation is accompanied by reduced autophagosome clearance, indicating a block in autophagy progression [126,127]. The activators of heme oxygenase-1 (HO-1), a protective enzyme with antioxidant and anti-inflammatory properties, alleviate lung injury by enhancing SQSTM1-mediated autophagic degradation of the NLRP3 inflammasome, thus limiting inflammation [126,128]. As inflammasome activation drives IL1 family cytokine release and NET formation, which in turn amplify cytokine storm during infection-associated lung injury [129], these observations support a broader role for SQSTM1 in limiting lung inflammation through selective autophagic clearance of NLRP3 components [14,62].

Although SQSTM1-mediated autophagy can restrain inflammation, its dysregulation may contribute to pulmonary injury. Oxidative stress induced by LPS or heme promotes the formation of SQSTM1-ubiquitin-positive aggresome-like induced structures (ALIS) in an iron-dependent manner, leading to toxic protein aggregation [58,130]. Inhibition of iron release by ferritin limits this process, highlighting the iron-sensitive nature of SQSTM1 aggregation.

**Figure 3:**
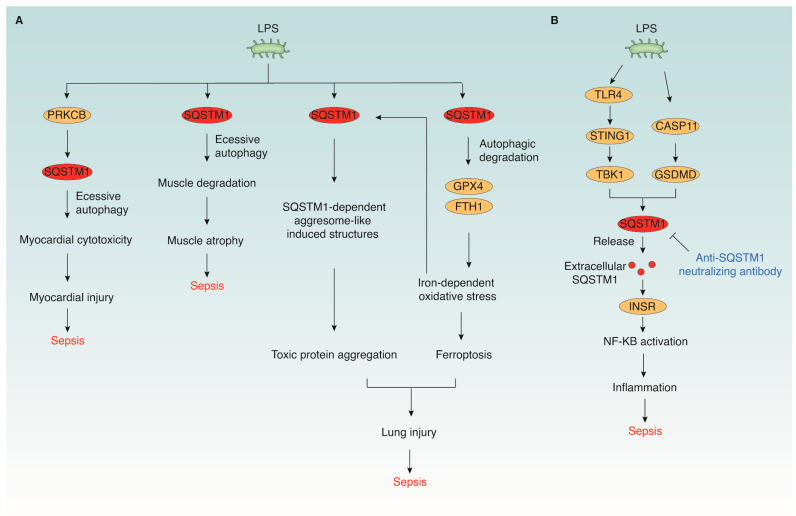
Pathogenic roles of SQSTM1 during lethal infection. Lipopolysaccharide (LPS) activates multiple signaling cascades in immune and parenchymal cells that converge on SQSTM1 to drive systemic inflammation and organ injury. (**A**) LPS serves as a trigger for intracellular SQSTM1-dependent injury across multiple tissues. In the heart, LPS-induced PRKCB activation drives excessive SQSTM1-dependent autophagy, resulting in myocardial cytotoxicity. In skeletal muscle, prolonged SQSTM1 activity leads to muscle degradation and atrophy. Lung injury is enhanced by the formation of SQSTM1-dependent aggresome-like induced structures (ALIS) and toxic protein aggregation. LPS also triggers SQSTM1-dependent autophagic degradation of ferroptosis suppressors such as GPX4 and ferritin heavy chain 1 (FTH1), promoting iron-dependent oxidative stress and ferroptotic cell death. (**B**) LPS stimulation via TLR4–STING1–TBK1 or CASP11–GSDMD signaling promotes intracellular accumulation and release of SQSTM1 through secretory or lytic pathways. Extracellular SQSTM1 engages the insulin receptor (INSR) on immune cells, activating NF-κB signaling and driving pro-inflammatory cytokine production. The anti-SQSTM1 neutralizing antibody confers protection against excessive inflammation by modulating extracellular SQSTM1 activity. Together, these processes converge to exacerbate inflammation and organ dysfunction, contributing to the development of sepsis.

Additionally, LPS triggers ferritinophagy in MLE-12 cells and septic mouse lungs, a process that depends on SQSTM1-mediated delivery of ferritin, the iron storage protein, to autophagosomes [105,131]. The subsequent ferritin degradation increases labile iron, leading to ROS generation, lipid peroxidation, and ferroptosis. This non-apoptotic, iron-dependent form of cell death has emerged as a key contributor to ALI [132,133,134], and ferroptotic epithelial death further aggravates barrier dysfunction. Genetic inhibition of *Sqstm1* limits ferritin degradation and mitigates ferroptotic damage, indicating a pathogenic role for SQSTM1 in promoting ferroptosis during lung injury. Conversely, hydrogen sulfide administration attenuates ferroptosis by upregulating glutathione peroxidase 4 (GPX4) and suppressing SQSTM1-dependent autophagy [103,135] (Figure 3). These findings emphasize the dual nature of SQSTM1: while essential for homeostatic autophagy, its unchecked activity may facilitate iron-driven oxidative injury in septic lungs.

Several pharmacologic and natural agents have demonstrated efficacy in modulating SQSTM1-dependent pathways to confer pulmonary protection. For instance, resveratrol enhances SQSTM1-mediated mitophagy and ameliorates mitochondrial dysfunction through phospholipid scramblase 3 (PLSCR3) regulation in CLP-induced ALI [102]. Ketamine promotes SQSTM1-autophagy and decreases apoptosis in LPS-exposed lung tissues and alveolar epithelial cells [104]. Similarly, compounds such as carvacrol, salidroside, and malvidin restore autophagic flux and suppress SQSTM1 accumulation, leading to reduced pulmonary inflammation and tissue damage [86,93,136]. In contrast, miR-210-3p—delivered via plasma extracellular vesicles—exacerbates ALI by targeting ATG7, thereby inhibiting autophagy and promoting inflammation [137] (Table 1).

Together, SQSTM1 serves as both a critical anti-inflammatory effector and a molecular rheostat whose dysregulation is central to the inflammatory cascade and subsequent tissue damage in infection-induced lung failure. However, it remains unclear how SQSTM1 differentially regulates protective versus deleterious autophagy pathways during the transition from early to late stages of ALI. Moreover, the precise molecular crosstalk between SQSTM1-mediated ferritinophagy and ferroptosis in human ARDS patients remains poorly defined and warrants further investigation.

### 5.5. Muscle

Infection frequently leads to skeletal muscle wasting, characterized by a progressive decline in muscle mass and strength, which contributes to long-term morbidity, ICU-acquired weakness, and increased mortality in survivors [138]. The underlying mechanisms include excessive proteolysis, mitochondrial dysfunction, and sustained inflammation. Autophagy plays a critical homeostatic role in muscle tissue by removing damaged proteins and organelles, but becomes dysregulated under septic conditions [139].

In a rat model of CLP-induced infection, autophagic degradation of SQSTM1 initially increases at 4 h but is markedly suppressed by 24 h in the anterior tibial muscle [140]. Pharmacologic induction of autophagy with rapamycin improves muscle function and survival by day 7, whereas inhibition with 3-MA exacerbates inflammation and impairs muscle recovery. These findings suggest that early enhancement of autophagy is protective against infection-induced myopathy, while impaired autophagic flux may contribute to persistent muscle dysfunction.

Nutritional and pharmacologic strategies that restore autophagic activity have shown therapeutic potential. Administration of alpha-lipoic acid (ALA) enhances autophagic flux, reduces SQSTM1 accumulation, and improves muscle structure and function in CLP-treated rats [141]. Similarly, exogenous activation of PRKAA1 signaling using AICAR reduces SQSTM1 levels and suppresses catabolic signaling, thereby preserving muscle mass and function [142] (Table 1).

However, the role of SQSTM1 in skeletal muscle during infection appears to be context-dependent and at times paradoxical. In both CLP- and LPS-induced mouse models, skeletal muscle exhibits activated autophagy accompanied by reduced expression of SQSTM1 and DNA damage-inducible transcript 4 (DDIT4), a negative regulator of MTOR signaling. Genetic deletion of *Ddit4* preserves protein synthesis and suppresses autophagy activation, resulting in increased SQSTM1 accumulation [142]. During the recovery phase, the AKT–MTOR pathway-driven protein synthesis is restored, whereas suppressed autophagic activity—evidenced by increased MAP1LC3 and SQSTM1 levels—coincides with anabolic signaling [143]. These results underscore a complex regulatory landscape in which SQSTM1 functions both as an autophagy receptor and as a potential mediator of anabolic signaling during muscle remodeling.

Emerging evidence also implicates SQSTM1 in mitochondrial quality control. For example, neuregulin 1 (NRG1) attenuates infection-induced muscle atrophy by inhibiting SQSTM1-mediated autophagy via the AKT–MTOR pathway, suggesting that excessive or prolonged SQSTM1-driven autophagy may shift from a protective mechanism to one that promotes muscle degradation [97]. This dual role of SQSTM1 highlights the importance of maintaining a balance in autophagic activity to support muscle homeostasis.

Together, these findings suggest that SQSTM1 plays dual roles in septic muscle—regulating both proteolysis and protein synthesis—where its accumulation marks autophagy impairment and its dynamic control influences muscle wasting and recovery.

### 5.6. Brain

Infection can cause sepsis-associated encephalopathy (SAE), a diffuse brain dysfunction marked by cognitive impairment, neuroinflammation, and neuronal apoptosis [144]. In CLP-induced murine models of infection, autophagy is activated in brain tissues—particularly in the cerebral cortex and hippocampal microglia—within 6 to 96 h, as evidenced by decreased SQSTM1 expression [99,145,146]. Pharmacologic inhibition of autophagy 3-MA attenuates behavioral deficits, suppresses microglial activation, and reduces neuroinflammation, whereas rapamycin exacerbates these phenotypes [147]. Similarly, CLP induces nuclear autophagy and SQSTM1 degradation in SAE, while genetic ablation of *Gsdmd* reverses these changes and ameliorates behavioral impairment [148], suggesting that autophagy contributes to SAE pathogenesis.

Paradoxically, SQSTM1 accumulation has also been associated with impaired lysosomal degradation and neuronal injury, while enhancing SQSTM1 clearance appears neuroprotective. For example, rapamycin-mediated activation of autophagy alleviates cognitive deficits in CLP-treated mice by promoting SQSTM1 degradation [149]. Similarly, ginsenoside Rg1 and dexmedetomidine have been shown to reduce SQSTM1 accumulation and protect neurons by stimulating autophagy and antioxidant responses [100,150]. The long non-coding RNA Lethe improves cortical neuronal survival during infection by promoting SQSTM1 degradation through enhanced autophagic flux [99] (Table 1).

These observations point to a complex, bidirectional role for SQSTM1 in the septic brain—where both its autophagic depletion and pathological accumulation can disrupt neuronal integrity—highlighting the brain’s heightened vulnerability to autophagy imbalance during infection.

## 6. Extracellular SQSTM1 as DAMP

Although SQSTM1 is widely recognized as a multifunctional intracellular adaptor linking autophagy and inflammation, emerging evidence highlights its pathogenic role as a DAMP in the extracellular space during infection. Circulating levels of SQSTM1 are elevated in septic patients, as shown in both platelets and peripheral blood mononuclear cells (PBMCs), suggesting systemic release from activated or damaged cells [75,76,110].

Recent mechanistic studies have elucidated that SQSTM1 can be actively secreted via non-classical pathways or passively released from LPS-stimulated myeloid cells undergoing stress or death. Extracellular SQSTM1 binds the insulin receptor (INSR) on myeloid cells, thereby triggering glycolytic reprogramming and activating NF-κB-dependent transcription of pro-inflammatory cytokines such as TNF, IL6, and IL1B [72] (Figure 3). In vivo, administration of recombinant mouse SQSTM1 (rSQSTM1) to wild-type mice induces systemic symptoms resembling septic shock—including lethargy, piloerection, and death—whereas these responses are abrogated in mice with myeloid cell-specific deletion of *Insr*. This finding establishes the pathogenic role of the extracellular SQSTM1–INSR axis in driving systemic inflammation and lethality.

Clinical relevance is further supported by data from a cohort of 40 septic patients, in which elevated plasma SQSTM1 levels positively correlate with both *SQSTM1* and *INSR* expression in PBMCs, as well as with clinical indicators of sepsis severity [72]. These results implicate secreted SQSTM1 as a functional DAMP and a driver of hyperinflammation and extracellular-mediated tissue injury during infection. Nevertheless, the evidence is derived from a relatively small cohort, highlighting the need for validation in larger, multicenter clinical studies.

Therapeutic strategies targeting extracellular SQSTM1 show promise. In murine models of bacterial infection, administration of anti-SQSTM1 neutralizing antibodies confers protection by reducing inflammatory cytokine release, apoptosis, disseminated intravascular coagulation (DIC), and overall mortality [72]. Additionally, synthetic ligands that target the ZZ-type zinc finger domain of SQSTM1—thereby promoting its self-polymerization and potentially preventing its interaction with pro-inflammatory receptors—effectively suppress IL1B, IL18, and IL6 release and improve survival in LPS-induced acute inflammation models [114]. These results suggest that modulating extracellular SQSTM1 activity represents a viable therapeutic avenue.

Collectively, these findings identify extracellular SQSTM1 as a critical DAMP that orchestrates metabolic reprogramming and potentiates innate immune activation during infection. Therapeutic targeting of this non-canonical, extracellular function of SQSTM1 represents a promising strategy to attenuate cytokine-mediated organ dysfunction and improve outcomes in septic patients. However, extracellular SQSTM1 may not be specific to infection, as it has also been implicated in sterile inflammatory conditions and neurological disorders such as acute pancreatitis and Huntington’s disease [74,151], which should be considered in future biomarker development.

## 7. Conclusions and Outlook

**Figure 4:**
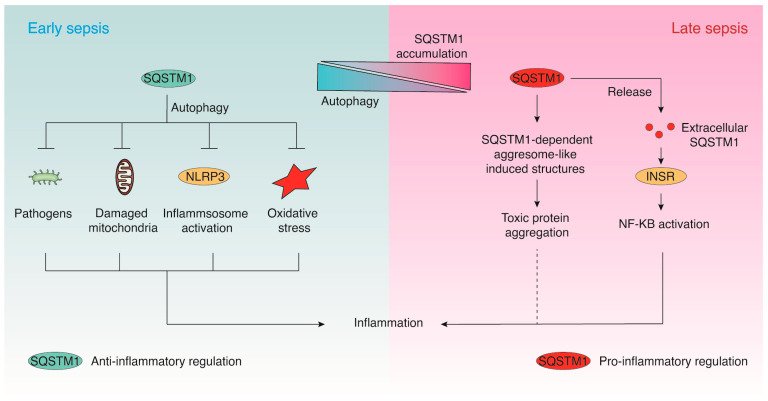
Conceptual model of stage-specific roles of SQSTM1 in lethal infection. (**Left**): In early sepsis, intracellular SQSTM1 supports cell survival by promoting autophagy, thereby limiting inflammasome activation and cytokine release. (**Right**): In late sepsis, impaired autophagic flux leads to SQSTM1 accumulation and SQSTM1 is released outside the cell. Intracellular SQSTM1 accumulation leads to the formation of SQSTM1-dependent aggresome-like induced structures (ALIS), which are associated with enhanced inflammatory responses and immune activation. Extracellular SQSTM1 acts as a damage-associated molecular pattern (DAMP), engaging receptors such as INSR to drive cytokine storm and multiorgan injury. This model highlights the dual, context-dependent functions of SQSTM1 during infection.

SQSTM1 is increasingly recognized as a central integrator of cellular stress responses, bridging autophagy, proteostasis, and inflammation across diverse physiological and pathological settings. In the context of infection, despite organ-specific differences, a unifying pattern emerges: intracellular SQSTM1 supports protective autophagic homeostasis in early infection, whereas impaired autophagic flux and SQSTM1 accumulation are consistently associated with metabolic stress, inflammasome activation, and tissue injury (Figure 4). SQSTM1 thus exerts context-dependent roles—facilitating protective autophagic clearance under certain conditions while promoting pathological inflammation or cell death when dysregulated. The discovery of SQSTM1 as an extracellular DAMP that activates INSR-mediated pro-inflammatory signaling introduces a new paradigm in our understanding of sterile inflammation and immunometabolic dysregulation during lethal infection.

Despite significant progress, several challenges remain. First, the dual roles of SQSTM1—as a pro-survival autophagy receptor versus a pro-inflammatory signal—are not yet fully delineated. It remains unclear how different stress cues, cell types, or stages of disease determine the functional outcome of SQSTM1 signaling. Second, the mechanisms regulating SQSTM1 secretion, its receptor specificity (e.g., INSR vs. AGER), and the downstream signaling consequences in various immune and parenchymal cells are not completely understood. Among the currently defined pathways, extracellular SQSTM1–INSR signaling appears to engage NF-κB activation and glycolysis-dependent inflammatory macrophage reprogramming; however, its broader links to immune checkpoint regulation and late-phase immunosuppression remain unclear. Third, many studies rely on bulk tissue or cell population-level analyses; the heterogeneity of SQSTM1 functions across cell types and subcellular compartments warrants single-cell and spatial-resolution studies.

From a translational standpoint, targeting SQSTM1 poses both opportunity and complexity. Strategies that enhance autophagic flux may restore intracellular SQSTM1 homeostasis but could inadvertently promote extracellular release. Conversely, neutralizing extracellular SQSTM1 or blocking its receptor interactions may suppress inflammation but risk impairing beneficial stress responses. The development of targeted therapeutics will require a nuanced understanding of the temporal dynamics, compartmental localization, and post-translational modifications of SQSTM1 during infection. In addition to SQSTM1 itself, the downstream therapeutic directions might be worth pursuing, including inhibition of the INSR-linked inflammatory cascade, particularly the NF-κB pathway, as well as modulation of glycolysis-associated immunometabolic reprogramming in macrophages. Although immune checkpoint pathways such as PD-1/PD-L1 are highly relevant to sepsis-associated late immunosuppression, their mechanistic linkage to SQSTM1–INSR signaling remains to be further established.

Future research should prioritize (1) defining the structural determinants of SQSTM1’s interaction with distinct receptors and cargoes, (2) integrating single-cell and spatial transcriptomic approaches to dissect SQSTM1 heterogeneity across tissues and disease stages, (3) establishing longitudinal monitoring of extracellular SQSTM1 levels in septic patients to validate its clinical utility as a biomarker, and (4) developing SQSTM1-targeting strategies, including small-molecule modulators, neutralizing antibodies, and spatially selective delivery approaches, to preserve beneficial intracellular SQSTM1-dependent homeostatic functions while limiting extracellular SQSTM1-driven inflammatory signaling.

In conclusion, SQSTM1 represents a compelling molecular hub with diagnostic, prognostic, and therapeutic potential in inflammation and infection. Advancing our mechanistic and translational understanding of its dual roles—both intracellular and extracellular—could inform novel strategies to modulate inflammation and preserve organ function in critically ill patients.

## Figures and Tables

**Table 1:** Therapeutic interventions targeting SQSTM1 in inflammation and infection.

Tissue	Experimental Model	SQSTM1 Function	Autophagic Regulation	Pathological Effects	Agent and Its Category	Agent Outcomes	Translational Note	Refs
Liver	CLP-induced sepsis mice; primary/cultured hepatocytes	Downregulation of SQSTM1 linked to impaired hepatocyte survival in sepsis	Inhibited autophagic flux worsens hepatocellular damage	Hepatic inflammation, apoptosis, mitochondrial injury	Genipin, Autophagy inducer	Genipin restored autophagy and ameliorated liver damage in sepsis	Natural compound; preclinical evidence only	[78]
Liver	LPS-induced acute liver injury mouse model	SQSTM1 decrease associated with aggravated NLRP3-driven inflammation	Autophagy inhibition exacerbates injury	Severe hepatocellular necrosis, oxidative stress	Malvidin, Antioxidant; anti-inflammatory (anthocyanin)	Malvidin activates NFE2L2, inhibits NLRP3, reduces apoptosis via autophagy	Dietary flavonoid; translational potential; no sepsis clinical data	[86]
Liver	CLP-induced liver injury mouse model	Downregulation of SQSTM1 linked to impaired hepatocyte survival in sepsis	Autophagy inhibition inflammation	Inflammation and oxidative injury	GTS-21, α7 nAChR agonist; anti-inflammatory; pro-autophagy	Promotes autophagy via α7 nAchR pathway; reduces inflammation	Experimental compound; limited clinical data in sepsis	[81]
Liver	CLP-induced septic liver injury mouse model	Suppressed SQSTM1-mediated flux contributes to hepatocellular death	MTOR pathway inhibits SQSTM1 function	Massive hepatocyte apoptosis	Anemoside B4,	Promotes autophagy via MTOR suppression; improves liver function	Herbal-derived compound; preclinical evidence	[80]
Kidney	LPS-induced SA-AKI mouse model; renal tubular epithelial cells	Increased intracellular SQSTM1 expression in tubular epithelial cells during early AKI	Delayed autophagosome clearance leads to autophagic stress	Acute tubular necrosis, cell death	Necrostatin-1, Necroptosis inhibitor; promotes autophagosome clearance	Promotes autophagosome elimination and improves renal function	Tool compound; not clinically established	[87]
Kidney	LPS-induced AKI mouse model; HK-2/HEK-293 cells	Cytoplasmic SQSTM1 upregulated under septic stress	PRKAA1-SIRT1 pathway stimulates autophagy	Tubular apoptosis, oxidative stress	rhEPO, Erythropoietin; anti-inflammatory; autophagy activator	Activates protective autophagy, reduces apoptosis via PRKAA1-SIRT1	Clinically available for anemia; sepsis renal use investigational	[87]
Kidney	CLP-induced SA-AKI rat model; HK-2 cells	SQSTM1 dysfunction impairs mitophagy via PRKN acetylation	SIRT7 activation enhances mitophagy	Reduced kidney inflammation and injury	Zn^2+^, mitophagy enhancer (PRKN acetylation via SIRT7)	SIRT7-mediated PRKN acetylation alleviates AKI	Supplement; dosing/benefit in sepsis requires validation	[88]
Kidney	Sepsis-AKI mouse models; HK-2 cells	SQSTM1 involved in autophagy-mediated tubular repair	BMSC-exosomes stimulate SQSTM1 signaling	AKI resolution and improved tubular function	BMSC-exosomes, Cell-free exosome therapy; pro-autophagy; anti-inflammatory	Exosomes reduce inflammation and promote autophagy in sepsis-AKI	Experimental biological therapy; preclinical evidence only	[89]
Kidney	CLP-induced AKI mouse model	SQSTM1 regulated through SIRT6-mediated deacetylation	Enhanced mitophagy and stress response	Decreased kidney inflammation	Polydatin, Antioxidant; SIRT6 activator; autophagy regulator	Activates SIRT6, promotes autophagy, reduces renal injury	Natural derivative of resveratrol; preclinical evidence	[90]
Heart	LPS-treated H9C2 cardiomyocytes	SQSTM1 deficiency aggravates cardiac oxidative stress	Controlled autophagy alleviates cardiac inflammation	Septic cardiomyopathy	Remifentanil, Opioid analgesic; PKCβ2 inhibitor; autophagy suppressor (harmful autophagy)	Downregulates PKCβ2 and inhibits harmful autophagy in cardiomyocytes	Clinically used analgesic; cardioprotective role preclinical	[91]
Heart	LPS-induced cardiomyocyte injury (H9C2)	SQSTM1 regulates apoptotic pathways via SIRT1-MTOR axis	Protective autophagy attenuates myocardial injury	Cardiomyocyte necrosis, inflammation	NaHS + miR-133a-3p, H_2_S donor plus miRNA; autophagy modulation via SQSTM1	miRNA modulates autophagy through SQSTM1 pathway	Experimental combination; preclinical evidence only	[92]
Heart	LPS-induced cardiomyopathy mouse model	SQSTM1-linked NLRP3 activation exacerbates pyroptosis	Autophagy suppression worsens cardiac function	Inflammation-driven cardiac injury	Carvacrol, Monoterpenoid; anti-inflammatory; anti-pyroptotic	Suppresses pyroptosis by inhibiting NLRP3-caspase1-GSDMD	Natural compound; preclinical evidence	[93]
Heart	LPS-induced cardiac injury models; H9C2 cells	SQSTM1 activation enhances BECN1 expression and autophagic clearance	Improved cardiac survival in sepsis	Reduced oxidative injury	Vitamin C, Antioxidant; autophagy inducer (BECN1)	Induces Beclin1-mediated autophagy and decreases inflammation	Clinically available vitamin; mixed clinical trial data in sepsis	[94]
Heart	CLP-induced myocardial injury mouse model	SQSTM1 mediates PRKAA1 activation and cardiac autophagy	Protective effect on cardiac tissue	Myocardial injury and inflammation	Semaglutide, GLP-1 analog; AMPK activator; pro-autophagy	Reactivates PRKAA1-SQSTM1 pathway, improves cardiac autophagic flux	Clinically approved antidiabetic; repurposing under investigation	[95]
Heart	CLP-induced cardiac injury mouse model	SQSTM1 accumulation regulated by ROS-NFE2L2 axis	Autophagy maintains cardiac integrity	Reduced fibrosis and inflammation	Thymoquinone, Antioxidant; supports SQSTM1-linked protective autophagy	Supports SQSTM1-linked autophagy to reduce cardiac injury	Natural compound; preclinical evidence only	[96]
Muscle	Sepsis-induced muscle atrophy rodent models	Decline in SQSTM1 contributes to excessive protein degradation	Uncontrolled autophagy worsens muscle atrophy	Skeletal muscle wasting, diaphragmatic dysfunction	Neuregulin-1β, Growth factor; inhibits excessive autophagy via AKT–MTOR	Inhibits autophagy via AKT-MTOR to prevent muscle loss	Biologic; experimental in sepsis	[97]
Muscle	Rodent sepsis models (skeletal muscle)	Mitophagy activation reduces LPS-induced injury	Autophagy promotes muscle cell survival	Decreased mitochondrial injury	Periplaneta americana extract, Traditional medicine extract; mitophagy activator (PINK1–PRKN–SQSTM1)	Activates PINK1–PRKN–SQSTM1 mitophagy axis	Traditional extract; preclinical evidence	[98]
Brain	Sepsis-associated encephalopathy (SAE) rodent model	Nuclear SQSTM1 accumulation linked to neuronal apoptosis	Dysregulated autophagy leads to neuronal death	Cognitive dysfunction in SAE	LncRNA Lethe, Long non-coding RNA; autophagy restoration	Restores autophagy and reduces cortical apoptosis in SAE model	Genetic modulation; experimental only	[99]
Brain	SAE rodent models; neuronal cells	SQSTM1 regulates non-canonical autophagy in CNS	SQSTM1–BECN1 axis drives protective autophagy	Ameliorates microglial inflammation	Ginsenoside Rg1, Traditional medicine; autophagy modulation; neuroprotection	Enhances neuroprotection via autophagy modulation	Herbal saponin; preclinical evidence	[100]
Brain	SAE rodent models	SIRT1-SQSTM1 axis supports neuron survival	Balanced autophagy decreases oxidative injury	Apoptosis inhibition in hippocampus	Melatonin, Antioxidant; SIRT1–SQSTM1 pathway activator	Activates SIRT1–SQSTM1 pathway, restores homeostasis	OTC hormone; clinical safety known; benefit in sepsis investigational	[101]
Lung	CLP-induced ALI/ARDS mouse model	SQSTM1 and MAP1LC3B elevated; suggest activated ferritinophagy	Excessive autophagy triggers ferroptosis	Acute lung inflammation and injury	Resveratrol, Antioxidant polyphenol; autophagy activator	Enhances mitophagy via PLSCR3, protects lung tissue	Dietary polyphenol; preclinical evidence; mixed clinical data	[102]
Lung	CLP-induced sepsis ALI mouse model; LPS-stimulated RAW264.7 and pulmonary epithelial cells	SQSTM1 loss disrupts MTOR–autophagy axis	Inflammation increased via ferroptosis	Pulmonary edema and damage	Hydrogen sulfide, Antioxidant and autophagy modulator	Inhibits MTOR, promotes autophagy, reduces ferroptosis	Experimental evidence only	[103]
Lung	CLP-induced sepsis (liver/lung) models; AEC II cells (in vitro)	SQSTM1 linked to impaired protection via PRKAA1-MTOR inhibition	Autophagy restoration improves outcomes	inflammation in alveolar type II epithelial cells	Ketamine, Anesthetic; AMPK–mTOR autophagy activator	Activates PRKAA1-MTOR to improve autophagy and survival	Clinically approved anesthetic; organ-protective potential in sepsis (preclinical)	[104]
Lung	LPS-induced MLE-12 lung epithelial cells and mouse sepsis models	YTHDC1–SQSTM1–ANGPTL4 axis modulates ferritinophagy	SQSTM1 overactivation induces cell death	Lung injury in late-stage sepsis	Mir22hg inhibition, lncRNA (MIR22HG) targeting; regulator of ferroptosis and ferritinophagy	Inhibits ANGPTL4 stabilization to reduce ferroptosis	Natural compound; preclinical evidence only	[105]

## Data Availability

No new data were created or analyzed in this study.
